# Impact of mean arterial pressure on reproductive endocrine characteristics in infertile patients with polycystic ovary syndrome: a secondary analysis of a randomized clinical trial

**DOI:** 10.3389/fendo.2025.1594813

**Published:** 2025-09-17

**Authors:** Baichao Shi, Yu Wang, Rong Luo, Yang Liu, Fengjuan Lu, Muxin Guan, Jiannan Yu, Zhuwei Gao, Xiaoke Wu

**Affiliations:** ^1^ Heilongjiang University of Chinese Medicine, Harbin, China; ^2^ The First Affiliated Hospital, Heilongjiang University of Chinese Medicine, Harbin, China; ^3^ Jiangxi Chest Hospital, Nanchang, China

**Keywords:** polycystic ovary syndrome, mean arterial pressure, insulin resistance, metabolic syndrome, hyperandrogenism

## Abstract

**Objective:**

This study aims to evaluate the association between mean arterial pressure (MAP) and anthropometric, metabolic, and endocrine parameters in Chinese infertile women with polycystic ovary syndrome (PCOS).

**Methods:**

A total of 1,000 PCOS subjects were enrolled in the clinical trial project of Acupuncture and Clomiphene in the treatment of PCOS infertility patients (PCOSAct). Of these, 998 patients were selected for this study. Linear trends and regression analyses were conducted to evaluate the association between MAP and anthropometric, metabolic, and endocrine parameters. Logistic regression was employed to estimate the association between MAP and risk of insulin resistance (IR), nonalcoholic fatty liver disease (NAFLD) and hyperlipidemia. The receiver operating characteristics (ROC) curve was used to determine the predictive value of the MAP for IR, NAFLD and hyperlipidemia.

**Results:**

Linear trends revealed that the MAP was positively associated with age, height, body weight, body mass index (BMI), waist circumference (WC), hip circumference (HC), waist-to-hip ratio (WHR), systolic blood pressure (SBP) and diastolic blood pressure (DBP), hirsutism score, and acanthosis nigricans score, fasting blood glucose (FBG), fasting insulin (FINS), the homeostatic model assessment for insulin resistance (HOMA-IR), low-density lipoprotein (LDL), triglycerides (TG), total cholesterol (TC), apolipoprotein B (ApoB), ApoB/apolipoprotein A1 (ApoA1) ratio, total testosterone (TT), and free androgen index (FAI), as well as the prevalence of IR, metabolic syndrome (MetS), NAFLD, and hyperlipidemia. Conversely, MAP was negatively correlated with the quantitative insulin sensitivity check index (QUICKI), high-density lipoprotein (HDL), sex hormone-binding globulin (SHBG), luteinizing hormone (LH), the LH/follicle stimulating hormone (FSH) ratio, and anti-Müllerian hormone (AMH). After adjusting for age and BMI, a significant linear relationship was observed between MAP and WC, WHR, hirsutism score, FBG, LDL, TG, TC, ApoB, and ApoB/ApoA1 ratio. Logistic regression analysis demonstrated that participants in the highest quartile (Q4) of MAP had no significantly higher odds ratios (OR) for IR, NAFLD and hyperlipidemia after adjusting for confounding factors. The ROC curve analysis indicated that the AUC_IR_ was 0.593 (95%CI: 0.557 ~ 0.629), with 85.9% sensitivity and 28.8% specificity at a cut-off value of 82.83, and the AUC_NAFLD_ was 0.621 (95%CI: 0.554 ~ 0.687), with 69.4% sensitivity and 53.5% specificity at a cut-off value of 87.17, and the AUC_hyperlipidemia_ was 0.555 (95% CI: 0.518 ~ 0.592), with 39.5% sensitivity and 70.00% specificity at a cut-off value of 90.83.

**Conclusion:**

Elevated MAP is associated with dysregulation of glucose and lipid metabolism and alterations in endocrine hormone levels. It may thus serve as a promising screening approach for IR-related conditions in patients with PCOS.

## 1 Introduction

Polycystic ovary syndrome (PCOS), a prevalent endocrine disorder affecting 5%–18% of reproductive-aged women (1), manifests through heterogeneous clinical features including hyperandrogenism (HA), insulin resistance (IR), menstrual irregularities, oligo-ovulation or anovulation, and infertility. This condition carries significant metabolic sequelae, notably heightened risks of early-onset type 2 diabetes mellitus (T2DM), pregnancy complications, cardiovascular disease (CVD), and endometrial cancer (2–4). Concurrently, PCOS is associated with psychological comorbidities—encompassing anxiety, depression, and body image disturbances—that collectively impair health-related quality of life across the lifespan (1, 4, 5).

Importantly, age and body mass index (BMI) matched women with PCOS demonstrate elevated systolic (SBP) and diastolic blood pressure (DBP) alongside increased hypertension prevalence compared to normal-weight controls (6), suggesting blood pressure dysregulation may contribute to PCOS pathophysiology. Although current guidelines recommend SBP/DBP assessment (7), emerging evidence positions mean arterial pressure (MAP)—reflecting integrated cardiac output and peripheral resistance during the cardiac cycle (8) —as a superior predictor of cerebrovascular damage and hemodynamic alterations (9, 10). This clinical relevance is underscored by prospective data indicating each 13-mmHg MAP elevation augments major cardiovascular events by 13% in T2DM cohorts (11), implying MAP may offer enhanced CVD risk stratification in PCOS beyond conventional metrics.

Pathophysiological links between PCOS and hemodynamics are further evidenced by correlations of free androgen index (FAI), total testosterone (TT), and sex hormone-binding globulin (SHBG) with blood pressure parameters (SBP/DBP) (12). Proposed mechanisms include α1-adrenergic desensitization and impaired renal artery reactivity, potentially disrupting renin-angiotensin-aldosterone system (RAAS) regulation (13) and establishing a vicious cycle wherein androgen excess directly modulates vascular tone while blood pressure dysregulation exacerbates RAAS-mediated metabolic dysfunction (14). Despite these insights, blood pressure profiling in PCOS remains underexplored, with most MAP studies focused on general CVD populations. To address this gap, we leveraged data from 1,000 PCOS patients in the Acupuncture and Clomiphene for Chinese Women with Polycystic Ovary Syndrome trial (PCOSAct): 1) compare baseline characteristics across MAP strata; and 2) evaluate MAP’s predictive value for IR, non-alcoholic fatty liver disease (NAFLD), and hyperlipidemia in this population.

## 2 Materials and methods

### 2.1 Design and target population

This secondary analysis utilized data from the PCOSAct trial (ClinicalTrials.gov: NCT01573858), conducted across mainland China (2011–2015). The parent study primarily assessed the efficacy of acupuncture versus clomiphene citrate on live birth rates in infertile PCOS patients. Participants comprised 1,000 women meeting *modified Rotterdam criteria* for PCOS diagnosis and literatures (15, 16), defined as ≥ 2 of the following: (a) oligo- or anovulation; (b) clinical/biochemical HA (hirsutism: modified Ferriman-Gallwey score in Chinese ≥ 5) (17, 18); (c) polycystic ovaries (≥ 12 antral follicles [2–9 mm] or ovarian volume ≥ 10 cm³). Diagnoses excluded alternate HA etiologies (congenital adrenal hyperplasia, Cushing’s syndrome, androgen-secreting tumors). Comprehensive methodological details—including design, eligibility criteria, and primary outcomes—are documented in prior publications (19, 20). Ethical approval was granted by the First Affiliated Hospital of Heilongjiang University of Chinese Medicine Ethics Committee (No. 2010HZYLL-010).

### 2.2 Data collection

#### 2.2.1 Anthropometric measurements

Data collected at baseline enrollment visits comprised the following: age (years), height (m), BMI = weight [kg]/height² [m²] and categorized as follows: underweight/normal weight: BMI < 24.0 kg/m²; overweight: BMI 24.0 to < 28.0 kg/m²; and obesity: BMI ≥ 28.0 kg/m² (21), waist circumference (cm), hip circumference (cm), waist-to-hip ratio (WHR = waist [cm]/hip [cm]), SBP (mmHg), DBP (mmHg), MAP = DBP + (SBP - DBP)/3 (22), hirsutism score (using the modified Ferriman-Gallwey score), acne score (using a standard acne lesion count diagram and definitions), and acanthosis nigricans score (identified by the presence of dark, thick, velvety, pigmented skin of the neck).

#### 2.2.2 Biochemical parameters

Following a 12-hour overnight fast on menstrual cycle day 3, venous blood samples were obtained during baseline visits and analyzed at the Heilongjiang University of Chinese Medicine core laboratory. Biochemical parameters were quantified using standardized methodologies: fasting blood glucose (FBG, mmol/L; hexokinase method, Maker Biotechnology, China) and fasting insulin (FINS, pmol/L; electrochemiluminescence immunoassay [ECLIA], Roche Diagnostics, Switzerland); lipid profiles including triglycerides (TG, mmol/L) and total cholesterol (TC, mmol/L; enzymatic colorimetry, Wako Diagnostics, Japan), HDL and LDL (mmol/L; direct assays), apolipoproteins A1 (ApoA1) and ApoB (g/L; polyethylene glycol-enhanced immunoturbidimetry, Maker Biotechnology, China); and reproductive hormones: total testosterone (TT, nmol/L) and SHBG (nmol/L; chemiluminescent immunoassays, Siemens Healthineers, Germany), free testosterone (FT, pg/mL; radioimmunoassay), luteinizing hormone (LH, mIU/mL), follicle-stimulating hormone (FSH, mIU/mL), estradiol (E2, pmol/L), and anti-Müllerian hormone (AMH, ng/mL) [ECLIA, Roche Cobas 6000-E601]. Additionally, IR was assessed using the homeostasis model assessment (HOMA-IR) calculated as: HOMA-IR = FINS (mIU/mL) × FBG (mmol/L)/22.5. IR was defined by a HOMA-IR value of ≥ 2.69 (23). Insulin sensitivity was evaluated via the quantitative insulin sensitivity check index (QUICKI): QUICKI = 1/[log (FINS, μU/mL) + log (FBG, mg/dL)]. FAI was calculated as: FAI = TT (nmol/L)/SHBG (nmol/L) ×100. NAFLD was determined according to prior physical examinations, with no additional specific assessments performed in this study.

HA was defined as a TT level ≥ 1.67 nmol/L (24). MetS was diagnosed per Chinese female criteria requiring ≥3 components: WC ≥ 85 cm; SBP ≥ 130 mmHg and/or DBP ≥ 85 mmHg; TG ≥ 1.7 mmol/L; HDL < 1.04 mmol/L; FBG ≥ 6.1 mmol/L (25). Hyperlipidemia was defined according to Chinese guidelines as serum TC ≥ 6.22 mmol/L, triglycerides ≥ 2.26 mmol/L, HDL-C < 1.04 mmol/L, or LDL-C ≥ 4.14 mmol/L (26).

### 2.3 Statistical analyses

Statistical analyses were performed using IBM SPSS Statistics (Inc., Chicago, IL, USA version 26.0). Continuous variables are presented as mean ± standard (SD) deviation and categorical variables as frequency (percentage). For continuous variables that follow a normal distribution, analysis of variance (ANOVA) is employed to determine group differences. When continuous variables violate normality, the Kruskal-Wallis H test is utilized to assess group differences. Categorical variables are evaluated for group differences using the χ² test. Linear regression was used to establish the correlations between the MAP and the characteristics of anthropometric and biochemical parameters. Multivariable logistic regression analysis was utilized to calculate the odds ratio (OR) with 95% confidence interval (CI) for the associations between the MAP and IR, NAFLD, or hyperlipidemia status (dependent variables, modeled separately). Receiver operating characteristic (ROC) curve analysis evaluated MAP’s predictive capacity for IR, NAFLD and hyperlipidemia, with area under the curve (AUC) quantification. Optimal MAP thresholds were derived by maximizing the Youden index (sensitivity + specificity - 1). Statistical significance was defined as *P* < 0.05.

## 3 Results

**Figure 1:**
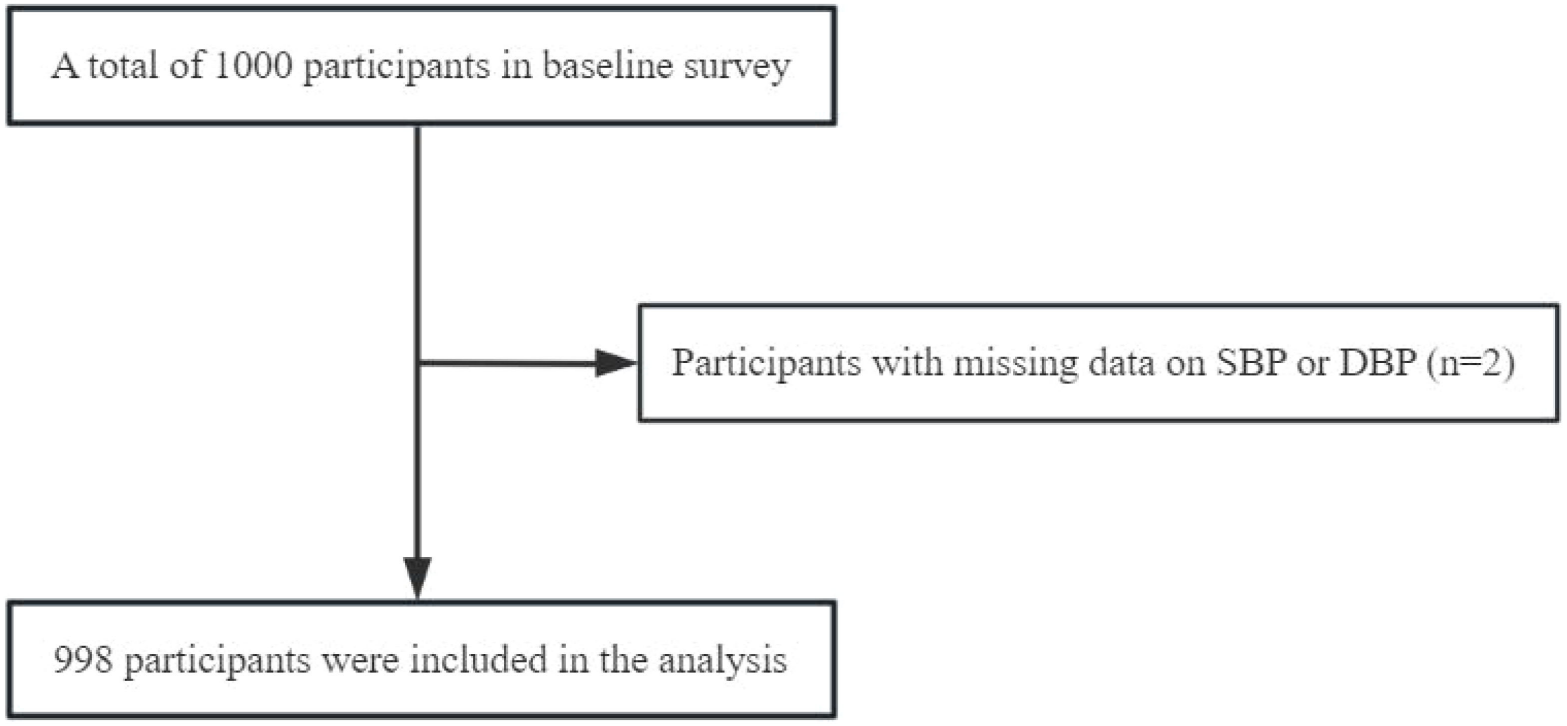
Flowchart of the study population.

This study included 998 patients with complete blood pressure data, constituting the analysis cohort (**Figure 1**). Participants were categorized into four groups based on MAP using the quartile method: Q1 (≤ 83.33 mmHg, n = 232), Q2 (83.34 - 86.67 mmHg, n = 282), Q3 (86.68- 93.33 mmHg, n = 187), and Q4 (≥ 93.34 mmHg, n = 297).

### 3.1 The baseline characteristics of the participants across the quartiles of MAP

As shown in **Table 1**, anthropometric indicators significantly increased across the MAP quartiles, including age, height, weight, BMI, WC, HC, WHR, SBP, DBP, hirsutism score and acanthosis nigricans score (*P*-trend < 0.05 for all). Specifically, the proportions of overweight and obesity increased across ascending MAP quartiles, while the proportion of underweight/normal weight decreased (*P*-trend < 0.05 for all). Regarding glucose and lipid metabolism, rising trends were observed for FBG, FINS, HOMA-IR, LDL, TG, TC, ApoB and ApoB/ApoA1ratio across the MAP quartiles (*P*-trend < 0.001 for all), while declining trends were noted for QUICKI and HDL (*P*-trend < 0.001 for all). In terms of biochemical indicators, TT, FAI, LH, LH/FSH ratio and AMH were increased (*P*-trend < 0.05 for all) and SHBG was decreased (*P*-trend < 0.001). The incidence of IR, MetS, NAFLD and hyperlipidemia exhibited a pronounced stepwise increase with ascending MAP quartiles (*P*-trend < 0.01 for all). However, there is no obvious linear trend relationship between MAP and acne score, ApoA1, FT, FSH and E2 (*P*-trend > 0.05 for all).

**Table 1:** Comprehensive clinical and biochemical characteristics of the included PCOS participants according to quartile of MAP.

Variables	Q1 (n=232)	Q2 (n=282)	Q3 (n=187)	Q4 (n=297)	*P*-value	*P* for trend
Anthropometric parameters						
Age, year, mean (SD)	27.55 (3.18)	28.01 (3.25)	27.56 (3.39)	28.35 (3.44)	0.017	0.014
Height, cm, mean (SD)	160.16 (5.15)	161.2 (5.11)	161.13 (4.76)	162.16 (5.06)	<0.001	<0.001
Weight, kg, mean (SD)	57.8 (9.98)	62.76 (11.26)	62.49 (12.28)	68.12 (13.41)	<0.001	<0.001
BMI, kg/m2, mean (SD)	22.52 (3.66)	24.11 (3.85)	23.97 (4.06)	25.85 (4.62)	<0.001	<0.001
Normal weight, n (%)	162 (69.83)	155 (54.96)	97 (51.87)	115 (38.72)	<0.001	<0.001
Overweight, n (%)	55 (23.71)	79 (28.01)	60 (32.09)	99 (33.33)	0.080	0.011
Obesity, n (%)	15 (6.47)	48 (17.02)	30 (16.04)	83 (27.95)	<0.001	<0.001
WC, cm, mean (SD)	80.96 (10.66)	84.55 (10.53)	85.22 (10.95)	89.89 (11.75)	<0.001	<0.001
HC, cm, mean (SD)	95.65 (7.52)	98.25 (8.33)	97.89 (8.96)	101.19 (8.72)	<0.001	<0.001
WHR, mean (SD)	0.84 (0.07)	0.86 (0.07)	0.87 (0.07)	0.89 (0.07)	<0.001	<0.001
SBP, mmHg, mean (SD)	101.22 (6.68)	110.38 (4.27)	114.99 (6.08)	121.08 (6.42)	<0.001	<0.001
DBP, mmHg, mean (SD)	65.9 (4.63)	71.37 (2.75)	77.55 (2.83)	83.48 (5.21)	<0.001	<0.001
Hirsutism score, mean (SD)	2.61 (2.54)	2.6 (2.63)	3.18 (2.95)	3.69 (2.94)	<0.001	<0.001
Acne score, mean (SD)	0.51 (0.79)	0.41 (0.77)	0.37 (0.68)	0.46 (0.77)	0.251	0.491
Acanthosis nigricans score, mean (SD)	1.12 (0.36)	1.18 (0.44)	1.21 (0.48)	1.31 (0.56)	<0.001	<0.001
Biochemical parameters						
FBG, mmol/L, mean (SD)	4.86 (0.82)	5.02 (0.8)	4.98 (1.02)	5.25 (1.18)	<0.001	<0.001
FINS, pmol/L, mean (SD)	77.38 (85.48)	94.62 (86.13)	95.51 (81.46)	112.43 (93.99)	<0.001	<0.001
HOMA-IR, mean (SD)	2.58 (3.79)	3.17 (3.46)	3.14 (2.90)	3.98 (3.97)	<0.001	<0.001
QUICKI, mean (SD)	0.35 (0.04)	0.34 (0.04)	0.34 (0.05)	0.33 (0.05)	<0.001	<0.001
HDL, mmol/L, mean (SD)	1.34 (0.37)	1.26 (0.34)	1.31 (0.43)	1.22 (0.35)	0.001	<0.001
LDL, mmol/L, mean (SD)	2.77 (0.79)	2.95 (0.86)	2.97 (1.03)	3.14 (0.82)	<0.001	<0.001
TG, mmol/L, mean (SD)	1.43 (0.86)	1.47 (0.8)	1.63 (1.06)	1.74 (0.92)	<0.001	<0.001
TC, mmol/L, mean (SD)	4.53 (0.99)	4.71 (1.07)	4.74 (1.29)	4.94 (1.01)	<0.001	<0.001
ApoA1, g/L, mean (SD)	1.52 (0.31)	1.5 (0.32)	1.51 (0.33)	1.5 (0.31)	0.893	0.525
ApoB, g/L, mean (SD)	0.81 (0.26)	0.9 (0.28)	0.89 (0.32)	0.98 (0.27)	<0.001	<0.001
ApoB/ApoA1ratio, mean (SD)	0.55 (0.18)	0.62 (0.20)	0.62 (0.21)	0.67 (0.20)	<0.001	<0.001
TT, nmol/L, mean (SD)	1.65 (0.61)	1.6 (0.64)	1.62 (0.62)	1.77 (0.68)	0.007	0.020
FT, pg/ml, mean (SD)	2.26 (0.86)	2.33 (0.84)	2.14 (0.83)	2.37 (0.82)	0.026	0.312
SHBG, nmol/L, mean (SD)	46.75 (30.14)	42.46 (26.7)	45.54 (34.74)	37.68 (30.67)	0.004	<0.001
FAI, mean (SD)	5.17 (4.18)	5.2 (3.68)	5.56 (4.39)	7.2 (4.99)	<0.001	<0.001
LH, mIU/mL, mean (SD)	12.02 (6.66)	9.67 (5.32)	10.28 (5.58)	10.23 (5.9)	0.001	0.018
FSH, mIU/mL, mean (SD)	6.18 (1.67)	5.99 (1.78)	6.21 (1.71)	6.05 (1.5)	0.426	0.302
LH/FSH ratio, mean (SD)	2.07 (1.57)	1.66 (0.95)	1.7 (0.92)	1.72 (0.96)	0.001	0.017
E2, pmol/L, mean (SD)	296.45 (372.62)	275.62 (377.83)	250.04 (185.26)	255.79 (271.21)	0.409	0.123
AMH, ng/mL, mean (SD)	13.09 (6.33)	11.56 (6.36)	11.82 (5.82)	11.65 (6.65)	0.028	0.014
The incidence of IR, MetS, NAFLD and hyperlipidemia						
IR, n (%)	60 (27.40)	118 (44.03)	77 (43.26)	148 (52.30)	<0.001	<0.001
MetS, n (%)	11 (4.93)	25 (9.29)	23 (12.99)	90 (31.69)	<0.001	<0.001
NAFLD, n (%)	10 (4.31)	12 (4.26)	17 (9.09)	33 (11.11)	0.002	<0.001
Hyperlipidemia, n (%)	80 (35.71)	111 (41.42)	75 (42.13)	142 (50.00)	0.013	0.001

BMI, body mass index; WC, waist circumference; HC, hip circumference; WHR, waist-to-hip ratio; SBP, systolic blood pressure; DBP, diastolic blood pressure; FBG, fasting blood glucose; FINS, fasting insulin; HOMA-IR, homeostatic model assessment-insulin resistance; QUICKI, quantitative insulin sensitivity check index; HDL, high-density lipoprotein; LDL, low-density lipoprotein; TG, triglycerides; TC, total cholesterol; ApoA1, apolipoprotein A1; ApoB, apolipoprotein B; TT, total testosterone; FT, free testosterone; SHBG, sex hormone-binding globulin; FAI, free androgen index; LH, luteinizing hormone; FSH, follicle-stimulating hormone; E2, estradiol; AMH, anti-Müllerian hormone; IR, insulin resistance; MetS, metabolic syndrome; NAFLD, non-alcoholic fatty liver disease.

Participants were stratified into four phenotypes based on IR and HA: Group A (with IR and HA, n = 224), Group B (with IR and without HA, n = 250), Group C (with HA and without IR, n = 213), Group D (without IR and HA, n = 262). Significant differences were observed across phenotypes in anthropometric indicators, metabolic profiles, endocrine parameters, and prevalence rates of MetS, NAFLD, and hyperlipidemia. Furthermore, across phenotypic subgroups, increasing quartiles of MAP were associated with progressive changes in anthropometric indicators, metabolic profiles, and endocrine parameters. The results are presented in **Supplementary Table S1** and **Supplementary Table S2**.

### 3.2 Linear regression analysis between the MAP and the clinical and biochemical characteristics of the PCOS patients

In patients with PCOS, there was a significant positive correlation between MAP and year, BMI, WC, HC, WHR, hirsutism score, acanthosis nigricans score, FBG, FINS, HOMA-IR, LDL, TG, TC, ApoB, ApoB/ApoA1 ratio, and FAI (*P* < 0.01 for all), while there was a significant inverse relationship between MAP and QUICKI, HDL, SHBG, LH, and LH/FSH ratio (*P* < 0.05 for all). After adjusting for age and BMI, the associations between MAP and the following parameters were notably attenuated, including HC, acanthosis nigricans score, FINS, HOMA-IR, QUICKI, HDL, TT, SHBG, FAI, LH, LH/FSH ratio, and AMH (as shown in **Table 2**).

**Table 2:** Linear associations between the MAP and clinical and biochemical parameters.

Variables	Crude			Adjusted^a^		
	β	*P*	95% CI	β	*P*	95% CI
Age (year)	0.08	0.009	1.31~9.24	–	–	–
BMI (kg/m^2^)	0.29	<0.001	10.49~15.80	–	–	–
WC (cm)^*^	0.29	<0.001	13.33~20.21	0.16	0.003	3.18~15.18
HC (cm)^*^	0.23	<0.001	15.04~25.66	-0.006	0.908	-9.32~8.28
WHR^*^	0.22	<0.001	15.17~26.50	0.11	0.001	4.04~16.58
Hirsutism score^*^	0.16	<0.001	1.03~2.32	0.15	<0.001	0.99~2.21
Acanthosis nigricans score^*^	0.13	<0.001	2.85~8.13	0.05	0.102	-0.44~4.88
FBG (mmol/L)^*^	0.11	0.001	1.63~5.88	0.07	0.028	0.25~4.37
FINS (pmol/L)^*^	0.18	<0.001	1.23~2.51	0.05	0.162	~0.21~1.25
HOMA-IR^*^	0.19	<0.001	1.11~2.24	0.06	0.107	-0.11~1.16
QUICKI^*^	-0.18	<0.001	-0.004~-0.002	-0.05	0.116	-0.002~0.000
HDL (mmol/L)^*^	-0.09	0.005	-4.04~-0.70	-0.01	0.847	-1.84~1.51
LDL (mmol/L)^*^	0.18	<0.001	2.94~6.14	0.12	<0.001	1.37~4.55
TG (mmol/L)^*^	0.17	<0.001	1.59~3.38	0.07	0.042	0.04~1.93
TC (mmol/L)^*^	0.16	<0.001	3.12~7.28	0.10	0.001	1.40~5.47
ApoB (g/L)^*^	0.23	<0.001	4.09~7.04	0.14	<0.001	1.73~4.86
ApoB/ApoA1ratio^*^	0.22	<0.001	3.59~6.41	0.11	0.003	0.84~3.95
TT (nmol/L)^*^	0.06	0.052	-0.01~2.27	0.05	0.112	-0.21~1.99
SHBG (nmol/L)^*^	-0.15	<0.001	-2.53~-1.05	-0.03	0.478	-1.09~0.51
FAI*	0.17	<0.001	1.06~2.29	0.07	0.051	-0.003~1.30
LH (mIU/mL)^*^	-0.07	0.026	-1.58~-0.10	0.01	0.745	-0.62~0.87
LH/FSH ratio^*^	-0.09	0.009	-1.94~-0.28	-0.004	0.905	-0.88~0.78
AMH (ng/mL)^*^	-0.08	0.019	-1.63~-0.15	-0.02	0.622	-0.91~0.55

a Adjusting for age and BMI; ^*^ Log-transformed.

### 3.3 Adjusted logistic regression analysis between the MAP quartiles and the risks of IR, NAFLD and hyperlipidemia

As shown in **Table 3**, logistic regression analyses evaluated the independent associations between MAP quartiles and metabolic complications in the PCOS cohort. For IR, both univariate and age-adjusted logistic regression revealed a clear association between increased MAP and the elevated risk of IR (*P*-trend < 0.001). For NAFLD risk, higher MAP quartiles demonstrated significantly elevated odds in univariate and age-adjusted models (*P*-trend < 0.01). Similarly, hyperlipidemia exhibited increased odds with higher MAP in and age-adjusted analyses (*P*-trend < 0.05). However, the association with IR, NAFLD and hyperlipidemia were fully attenuated after further adjustment for BMI (*P*-trend > 0.05).

**Table 3:** Adjusted OR (95% CI) for the associations between the MAP and the risk of IR, NAFLD and hyperlipidemia.

Variables	Q1 (n=232)	Q2 (n=282)	Q3 (n=187)	Q4 (n=297)	*P* for trend
Median	79.71	83.54	90.02	93.63	
IR					
Model 1	1.00 (Reference)	2.09 (1.42~3.06]	2.02 (1.33~3.07]	2.91 (1.99~4.24)	<0.001
*P*-values		<0.001	0.001	<0.001	
Model 2	1.00 (Reference)	2.10 (1.43~3.08)	2.02 (1.33~3.08)	2.94 (2.01~4.29)	<0.001
*P*-values		<0.001	0.001	<0.001	
Model 3	1.00 (Reference)	1.59 (1.03~2.44)	1.56 (0.97~2.50)	1.51(0.97~2.32)	0.148
*P*-values		0.035	0.066	0.067	
NAFLD					
Model 1	1.00 (Reference)	0.99 (0.42~2.33)	2.22 (0.99~4.97)	2.78 (1.34~5.76)	0.004
*P*-values		0.976	0.053	0.006	
Model 2	1.00 (Reference)	0.96 (0.41~2.26)	2.22 (0.99~4.97)	2.64 (1.27~5.50)	0.005
*P*-values		0.924	0.053	0.009	
Model 3	1.00 (Reference)	0.68 (0.28~1.65)	1.67 (0.72~3.85)	1.26 (0.57~2.76)	0.149
*P*-values		0.394	0.230	0.569	
Hyperlipidemia					
Model 1	1.00 (Reference)	1.27 (0.88~1.84)	1.31 (0.88~1.96)	1.80 (1.26~2.58)	0.013
*P*-values		0.196	0.189	0.001	
Model 2	1.00 (Reference)	1.22 (0.85~1.77)	1.31 (0.87~1.97)	1.69 (1.18~2.43)	0.036
*P*-values		0.282	0.194	0.004	
Model 3	1.00 (Reference)	0.99 (0.67~1.45)	1.08 (0.71~1.65)	1.09 (0.74~1.61)	0.941
*P*-values		0.958	0.728	0.664	

OR, odds ratio; CI, confidence interval; IR, insulin resistance; NAFLD, non-alcoholic fatty liver disease.

Model 1, adjusted for no confounding factor; Model 2, was adjusted for age; Model 3, was adjusted for age and BMI.

### 3.4 The predictive value of the MAP in detecting IR, NAFLD and hyperlipidemia

**Figure 2:**
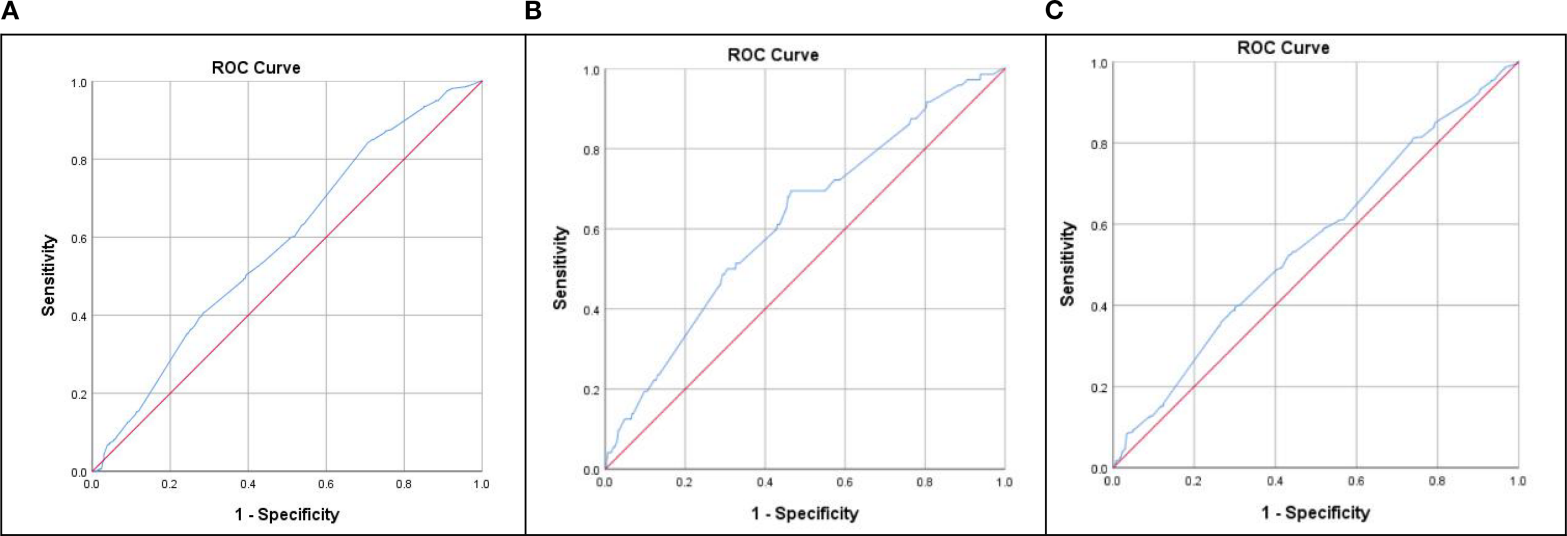
The results of ROC curve analysis regarding the predictability of MAP in IR **(A)**, NAFLD **(B)** and hyperlipidemia **(C)**.

In **Table 4** and **Figure 2**, panels A, B, and C displayed the ROC curves for MAP as a predictor of IR, NAFLD, and hyperlipidemia respectively. The ROC curve analysis of IR showed that the AUC was 0.593 (95% CI: 0.557 ~ 0.629), with a sensitivity of 85.9% and a specificity of 28.8%. The optimal cut-off value of the MAP for IR prediction was 82.83, and the Youden index was 0.147. For NAFLD, the MAP had a 69.4% sensitivity and 53.5% specificity with a threshold value of 87.17, the AUC was 0.621 (95%CI: 0.554 ~ 0.687), and the Youden index was 0.225. For hyperlipidemia, the AUC was 0.555 (95% CI: 0.518 ~ 0.592), with 39.5% sensitivity and 70.0% specificity at an optimal cut-off of 90.83 (Youden index: 0.230).

**Table 4:** The predictive value of MAP in detecting IR, NAFLD and hyperlipidemia.

Predictors	AUC (95% CI)	Cut-off value	Sensitivity	Specificity	Youden index	*P*-value
IR	0.593 (0.557~0.629)	82.83	85.9%	28.8%	0.147	<0.001
NAFLD	0.621 (0.554~0.687)	87.17	69.4%	53.5%	0.225	0.001
Hyperlipidemia	0.555 (0.518~0.592)	90.83	39.5%	70.0%	0.095	0.004

## 4 Discussion

Our study demonstrates elevated MAP is independently associated with adverse unfavorable anthropometric indicators, dysregulated glucolipid profiles, and heightened risks of IR, MetS, NAFLD, and hyperlipidemia in PCOS. Furthermore, MAP exhibits utility as a pragmatic clinical predictor for identifying individuals with IR, NAFLD, and hyperlipidemia in this population.

A core pathophysiological cornerstone of PCOS is IR, which intensifies hormonal dysregulation and anovulation, underpins metabolic abnormalities such as obesity and MetS, and significantly increases susceptibility to T2DM and CVD (2, 27–29). Notably, 65-95% of PCOS patients manifested IR with compensatory hyperinsulinemia (2), and those with concurrent IR exhibit atherogenic dyslipidemia characterized by elevated TG, LDL, ApoB, TG/HDL, and ApoB/ApoA ratio, with TG and ApoB levels exhibiting positive correlation with BMI (30). Previous studies established a strong correlation between blood pressure, particularly SBP, and FINS in childhood, with hypertensive children exhibiting higher adiposity, FINS, BMI, TC, and HDL-C levels, indicating that early-life blood pressure dysregulation may predispose individuals to glucolipid metabolic abnormalities in adolescence or reproductive-age PCOS (31). Our findings demonstrated that patients with higher quartile of MAP exhibit increased weight, BMI, WC, HC, WHR, and elevated levels of FBG, FINS, HOMA-IR, LDL, TG, TC, ApoB, as well as a superior ApoB/ApoA1 ratio. Additionally, elevated MAP is associated with a higher prevalence of IR and its related disorders, including MetS, NAFLD, and hyperlipidemia. In our study, MAP was further positively associated with WC, WHR, FBG, LDL, TG, TC, ApoB, and the ApoB/ApoA1 ratio, independent of age and BMI. M Sohlman et al. (32) reported that IR and/or hyperinsulinemia independently predict aortic stenosis, irrespective of other CVD risk factors (including diabetes, obesity, and SBP), implying a systemic connection between vascular function (such as blood pressure) and IR. Mechanistically, despite significantly impaired insulin-mediated glucose uptake via receptor substrate 1 (IRS1) in adipocytes under IR conditions with compensatory hyperinsulinemia, renal insulin signaling through IRS2 remains intact, promoting sodium reabsorption and contributing to volume expansion and hypertension (33). Concurrent hyperinsulinemia drives vascular dysfunction by altering vascular smooth muscle reactivity to increase peripheral resistance (34), and activating the renin-angiotensin-aldosterone system (RAAS) via endothelial angiotensin-converting enzyme (ACE)/angiotensin II (ANGII)/angiotensin receptor (AT1R) axis overstimulation while enhancing sympathetic nervous system (SNS) tone (35), collectively elevating MAP through hemodynamic alterations (36, 37); this is compounded by IR-induced endothelial dysfunction disrupting the nitric oxide/endothelin-1 balance (38, 39). Despite mechanistic insights, the MAP-IR relationship remains inadequately explored in PCOS, where HA and unique metabolic features may modulate these pathways. High-quality studies should elucidate: insulin-RAAS crosstalk in hypertension pathogenesis and dynamic MAP fluctuations in PCOS.

HA is another major feature of PCOS. Excessive activation of androgens results in ovulatory disorders, menstrual irregularities, hirsutism, and acne, suggesting that excessive androgens are not merely a clinical characteristic of PCOS, but they also serve as a significant risk factor (40). Our findings revealed that PCOS patients with elevated MAP manifested more severe hirsutism and acanthosis nigricans. Moreover, significant positive correlations were observed between MAP and both TT and FAI, while a negative association emerged with SHBG. These associations became non-significant after adjustment for age and BMI, with the exception of FAI which approached significance (*P* = 0.051), suggesting that FAI——reflecting bioactive circulating androgens—might exhibit greater sensitivity than TT in indicating MAP variations. Previous studies corroborated FAI’s involvement in obesity-related metabolic disturbances (41), while elevated androgens were independently associated with increased SBP and DBP (12, 14). Mechanistically, androgens exert direct or indirect effects via SNS activation and renin-angiotensin system (RAS) stimulation, promoting vasoconstriction and sodium reabsorption (14, 42). This shifts the pressure-natriuresis relationship, elevating blood pressure (14, 42). Collectively, these studies suggest that excessive androgen secretion might contribute to metabolic dysregulation, with MAP emerging as a composite outcome of obesity, insulin metabolism, and androgen steroid activity. It is also worth noting that patients with a higher MAP were found to be older and taller – a novel observation which has not yet been incorporated into clinical PCOS diagnostics but is an interesting finding.

Our study identified a distinct reproductive phenotype in PCOS patients with elevated MAP, characterized by decreased LH and LH/FSH ratio. This profile indicates hypothalamic-pituitary-ovarian (HPO) axis dysregulation, where impaired gonadotropin releasing hormone (GnRH) pulse control reduces sensitivity to negative feedback (43, 44). This disrupts gonadotropin dynamics, increasing GnRH pulse frequency and preferential LH secretion over FSH - a hallmark of PCOS pathogenesis that stalls follicular development while stimulating ovarian androgen production (45). Nevertheless, the mechanisms by which MAP affects the HPO axis remain unclear, although an association between LH and CVD has been reported (46). We hypothesized that direct hemodynamic effects on hypothalamic-pituitary perfusion, activation of SNS/RAS pathways by blood pressure fluctuations, or circadian blood pressure abnormalities may disrupt the pulsatile secretion of LH, potentially mediating the impact of MAP on the HPO axis. Consistent with the reproductive phenotype in increased-MAP PCOS patients, we observed reduced AMH levels. AMH participates in follicular development and modulates hypothalamic-pituitary-gonadal axis activity, serving as a biomarker of the ovarian follicular pool (47). Emerging evidence suggested that AMH may influence cardiovascular physiology: obese patients demonstrated significant AMH-cardiometabolic risk associations (OR = 1.77, 95%CI: 0.95 ~ 3.31, *P* = 0.049) (48), while another study identified a linear relationship between serum AMH and morning blood pressure surge (OR = 1.24, 95%CI: 1.02 ~ 1.50, *P* = 0.033) (49). These findings were aligned with our observed inverse MAP-AMH relationship in PCOS, suggesting AMH fluctuations could potentially modulate blood pressure regulation through shared pathways affecting ovarian and cardiovascular systems.

While PCOS confers substantially elevated risks of metabolic dysfunction compared to the general population, the clinical significance of MAP within PCOS-specific metabolic pathophysiology remains underexplored. Our study demonstrated that the AUC_IR_ was 0.593 and had a sensitivity of 85.9% and specificity of 28.8% with a threshold of 82.83 mmHg and Youden index of 0.147. We calculated the ROC curve for MAP in relation to NAFLD in PCOS for the first time, and determined that a MAP of 87.17 mmHg was the cut-off for NAFLD (with a sensitivity of 69.4% and a specificity of 53.5%). Furthermore, our study found MAP for hyperlipidemia (AUC = 0.555), with an optimal diagnostic cut-off of 90.83 mmHg demonstrating 39.5% sensitivity and 70.0% specificity. These findings establish MAP as a viable non-invasive indicator for metabolic complication screening in PCOS. Given the suboptimal diagnostic performance of individual MAP thresholds, future studies should: 1) expand cohort sizes to enhance statistical power, 2) incorporate multidimensional clinical parameters for advanced multivariate modeling, and 3) develop integrated diagnostic algorithms combining MAP with complementary biomarkers. Additionally, longitudinal cohort studies are warranted to elucidate causal relationships between MAP trajectories and metabolic complication progression. Such evidence would enable precision interventions targeting early cardiometabolic risk mitigation, ultimately improving long-term PCOS outcomes.

This study’s principal strengths include a large, nationally representative cohort of Chinese women with PCOS and the novel comprehensive characterization of MAP associations with both metabolic and reproductive endocrine parameters. However, several limitations merit consideration: 1) NAFLD diagnosis lacked ultrasonographic or biopsy validation, potentially introducing selection bias and restricting sample size; 2) Absence of comparator groups (healthy controls or women with other gynecological disorders) prevents clarification of PCOS-specific MAP patterns; 3) The cross-sectional design, inherent to this secondary analysis of PCOSAct trial data, precludes causal inference regarding temporal relationships between MAP elevation and metabolic complications. Consequently, it remains indeterminate whether MAP elevation represents a predictor or consequence of PCOS progression.

## 5 Conclusion

In conclusion, our results suggested that elevated MAP independently associates with exacerbated adiposity, dysglycemia, and atherogenic dyslipidemia in Chinese women with PCOS. Our findings support MAP’s utility as a practical clinical biomarker for preliminary screening of IR, NAFLD, and hyperlipidemia in this population. Future validation studies should develop integrated multimarker diagnostic models and establish standardized MAP thresholds through prospective cohorts to optimize clinical implementation.

## Data Availability

The original contributions presented in the study are included in the article/**Supplementary Material**. Further inquiries can be directed to the corresponding authors.
