# Supplementary material for: Impact of mean arterial pressure on reproductive endocrine characteristics in infertile patients with polycystic ovary syndrome: a secondary analysis of a randomized clinical trial
Source: Front Endocrinol (Lausanne). 2025 Sep 17;16:1594813. doi: 10.3389/fendo.2025.1594813 (PMC12483876; doi:10.3389/fendo.2025.1594813)
Supplement: Supplementary file 1 [file Table1.docx]

**Supplemental Material**

This analysis presents the baseline characteristics, prevalence of metabolic syndrome (MetS), non-alcoholic fatty liver disease (NAFLD), and dyslipidemia across different polycystic ovary syndrome (PCOS) phenotypes. Additionally, it examines the impact of mean arterial pressure (MAP) on endocrine and metabolic profiles within phenotypic subgroups. Results are detailed in Supplementary Tables 1 and 2.

**Supplement Table 1**. Comprehensive clinical and biochemical characteristics of the included PCOS participants according to different subgroups

| Variables | A group（n=224） | B group（n=250） | C group（n=213） | D group（n=262） | *P*-value |
| --- | --- | --- | --- | --- | --- |
| Anthropometric parameters | | | | | |
| Age, year, mean (SD) | 27.69 (3.26) | 28.15 (3.48) | 27.74 (2.98) | 28.05 (3.42) | 0.511 |
| Height, cm, mean (SD) | 161.86 (5.09) | 161.67 (5.23) | 160.42 (5.12) | 160.75 (4.84) | 0.005 |
| Weight, kg, mean (SD) | 69.88 (12.58) | 67.94 (12.16) | 57.47 (9.08) | 56.83 (9.46) | <0.001 |
| BMI, kg/m2, mean (SD) | 26.58 (4.28) | 25.92 (3.93) | 22.32 (3.21) | 21.98 (3.41) | <0.001 |
| WC, cm, mean (SD) | 91.73 (11.28) | 89.83 (10.78) | 80.15 (9.55) | 79.97 (9.03) | <0.001 |
| HC, cm, mean (SD) | 102.64 (8.21) | 101.76 (8.35) | 94.31 (7.22) | 94.68 (6.88) | <0.001 |
| WHR, mean (SD) | 0.89 (0.06) | 0.88 (0.07) | 0.85 (0.08) | 0.84 (0.07) | <0.001 |
| SBP, mmHg, mean (SD) | 114.54 (8.39) | 113.27 (8.21) | 111.77 (10.36) | 110.14 (9.64) | <0.001 |
| DBP, mmHg, mean (SD) | 75.98 (7.45) | 75.43 (7.17) | 74.54 (7.87) | 73.69 (8.49) | 0.017 |
| MAP, mmHg, mean (SD) | 88.83 (6.98) | 88.04 (6.7) | 86.95 (8.02) | 85.84 (8.1) | <0.001 |
| Hirsutism score, mean (SD) | 3.28 (2.95) | 2.92 (2.83) | 3.07 (2.7) | 2.78 (2.57) | 0.118 |
| Acne score, mean (SD) | 0.45 (0.73) | 0.44 (0.79) | 0.46 (0.78) | 0.4 (0.77) | 0.892 |
| Acanthosis nigricans score, mean (SD) | 1.28 (0.52) | 1.3 (0.58) | 1.11 (0.33) | 1.14 (0.4) | <0.001 |
| Biochemical parameters | | | | | |
| FBG, mmol/L, mean (SD) | 5.46 (0.94) | 5.42 (0.87) | 4.63 (0.9) | 4.65 (0.82) | <0.001 |
| FINS, pmol/L, mean (SD) | 133.66 (67.88) | 154.09 (121.4) | 47.17 (19.89) | 46.24 (18.56) | <0.001 |
| HOMA-IR, mean (SD) | 5.49 (3.32) | 6.44 (6.18) | 1.59 (0.65) | 1.6 (0.67) | <0.001 |
| QUICKI, mean (SD) | 0.3 (0.02) | 0.3 (0.02) | 0.37 (0.04) | 0.37 (0.04) | <0.001 |
| HDL, mmol/L, mean (SD) | 1.25 (0.37) | 1.19 (0.34) | 1.38 (0.4) | 1.3 (0.35) | <0.001 |
| LDL, mmol/L, mean (SD) | 3.28 (1.02) | 3.01 (0.83) | 2.93 (0.79) | 2.7 (0.74) | <0.001 |
| TG, mmol/L, mean (SD) | 1.93 (1) | 1.94 (1.01) | 1.18 (0.62) | 1.23 (0.62) | <0.001 |
| TC, mmol/L, mean (SD) | 5.14 (1.25) | 4.81 (1.06) | 4.69 (0.97) | 4.39 (0.92) | <0.001 |
| ApoA1, g/L, mean (SD) | 1.53 (0.3) | 1.49 (0.32) | 1.54 (0.33) | 1.47 (0.3) | 0.042 |
| ApoB, g/L, mean (SD) | 1.03 (0.33) | 0.96 (0.28) | 0.84 (0.23) | 0.78 (0.22) | <0.001 |
| ApoB/ApoA1ratio, mean (SD) | 0.69 (0.22) | 0.66 (0.2) | 0.56 (0.18) | 0.54 (0.16) | <0.001 |
| TT, nmol/L, mean (SD) | 2.23 (0.53) | 1.2 (0.33) | 2.19 (0.45) | 1.2 (0.29) | <0.001 |
| FT, pg/ml, mean (SD) | 2.75 (0.74) | 1.98 (0.69) | 2.69 (0.83) | 1.85 (0.7) | <0.001 |
| SHBG, nmol/L, mean (SD) | 33.47 (26.5) | 33.88 (28.36) | 50.14 (31.56) | 53.05 (29.63) | <0.001 |
| FAI, mean (SD) | 9.34 (5.19) | 5.26 (3.37) | 6.04 (3.96) | 3.18 (2.45) | <0.001 |
| LH, mIU/mL, mean (SD) | 10.76 (4.92) | 8.32 (5.22) | 13.71 (6.4) | 9.81 (5.89) | <0.001 |
| FSH, mIU/mL, mean (SD) | 5.93 (1.65) | 6.01 (1.72) | 6.26 (1.56) | 6.19 (1.69) | 0.206 |
| LH/FSH ratio, mean (SD) | 1.94 (1.39) | 1.4 (0.88) | 2.26 (1.11) | 1.64 (0.96) | <0.001 |
| E2, pmol/L, mean (SD) | 299.28 (406.86) | 223.04 (232.7) | 330.42 (356.3) | 240.58 (257.06) | 0.002 |
| AMH, ng/mL, mean (SD) | 12.52 (6.38) | 9.84 (5.08) | 14.63 (6.51) | 11.78 (6.66) | <0.001 |
| The incidence of IR, MetS, NAFLD and hyperlipidemia | | | | | |
| MetS, n (%) | 63 (28.13) | 59 (23.79) | 12 (5.69) | 13 (4.98) | <0.001 |
| NAFLD, n (%) | 25 (11.26) | 35 (14.11) | 6 (2.88) | 3 (1.17) | <0.001 |
| Hyperlipidemia, n (%) | 124 (55.36) | 137 (55.47) | 67 (31.60) | 88 (29.33) | <0.001 |

Three phenotypes were created using insulin resistance (IR) and hyperandrogenism (HA): (A) IR and HA, (B) without HA but with IR, (C) without IR but with HA, and (D) without IR and HA.

BMI, body mass index; WC, waist circumference; HC, hip circumference; WHR, waist-to-hip ratio; SBP, systolic blood pressure; DBP, diastolic blood pressure; MAP, mean arterial pressure; FBG, fasting blood glucose; FINS, fasting insulin; HOMA-IR, homeostatic model assessment-insulin resistance; QUICKI, quantitative insulin sensitivity check index; HDL,high-density lipoprotein; LDL, low-density lipoprotein; TG, triglycerides; TC, total cholesterol; ApoA1, apolipoprotein A1; ApoB, apolipoprotein B; TT, total testosterone; FT, free testosterone; SHBG, sex hormone-binding globulin; FAI, free androgen index; LH, luteinizing hormone; FSH, follicle-stimulating hormone; E2, estradiol; AMH, anti-Müllerian hormone; MetS, metabolic syndrome; NAFLD, non-alcoholic fatty liver disease.

**Supplement Table 2**. Comprehensive clinical and biochemical characteristics of the included PCOS participants according to quartile of MAP in different subgroups.

| Variables | A group（n=224） | | | | | | B group（n=250） | | | | | | C group（n=213） | | | | | | D group（n=262） | | | | | |
| --- | --- | --- | --- | --- | --- | --- | --- | --- | --- | --- | --- | --- | --- | --- | --- | --- | --- | --- | --- | --- | --- | --- | --- | --- |
|  | Q1 | Q2 | Q3 | Q4 | *P* | *P* for trend | Q1 | Q2 | Q3 | Q4 | *P* | *P* for trend | Q1 | Q2 | Q3 | Q4 | *P* | *P* for trend | Q1 | Q2 | Q3 | Q4 | *P* | *P* for trend |
| Anthropometric parameters | | | | | | | | | | | | | | | | | | | | | | | | |
| Age, year, mean (SD) | 27.49 (3.33) | 27.48 (3.21) | 26.92 (2.94) | 28.22 (3.35) | 0.184 | 0.171 | 27.48 (3.14) | 28.23 (3.25) | 27.98 (3.72) | 28.53 (3.71) | 0.462 | 0.198 | 27.2 (2.79) | 27.54 (2.89) | 27.27 (2.88) | 28.64 (3.13) | 0.028 | 0.01 | 27.73 (3.34) | 28.6 (3.35) | 27.78 (3.6) | 28.02 (3.46) | 0.395 | 0.885 |
| Height, cm, mean (SD) | 161.01 (4.98) | 161.66 (5.81) | 162.06 (5.03) | 162.27 (4.67) | 0.631 | 0.202 | 159.49 (5.82) | 161.74 (4.94) | 161.73 (4.9) | 162.67 (5.19) | 0.019 | 0.006 | 159.14 (4.95) | 160.4 (5.03) | 160.43 (4.48) | 161.57 (5.49) | 0.069 | 0.01 | 160.8 (5.07) | 160.67 (4.78) | 159.99 (4.27) | 161.64 (5.14) | 0.383 | 0.631 |
| Weight, kg, mean (SD) | 64.44 (9.54) | 68.11 (11.32) | 70.96 (11.16) | 72.88 (14.16) | 0.003 | <0.001 | 63.2 (11.57) | 66.94 (11.84) | 67.24 (11.93) | 71.86 (11.97) | 0.002 | <0.001 | 54.84 (9.26) | 56.04 (8.44) | 56.96 (6.44) | 61.18 (9.63) | <0.001 | <0.001 | 54.24 (7.05) | 57.78 (8.06) | 54.87 (9.32) | 61.94 (12.65) | <0.001 | <0.001 |
| BMI, kg/m2, mean (SD) | 24.82 (3.27) | 26.04 (3.95) | 26.84 (3.26) | 27.58 (4.93) | 0.006 | 0.001 | 24.77 (4.04) | 25.51 (3.86) | 25.64 (3.81) | 27.11 (3.79) | 0.008 | 0.001 | 21.65 (3.4) | 21.77 (2.54) | 22.16 (2.68) | 23.44 (3.5) | 0.006 | 0.001 | 20.98 (2.53) | 22.39 (3.13) | 21.4 (3.21) | 23.69 (4.49) | <0.001 | <0.001 |
| WC, cm, mean (SD) | 86.88 (10.59) | 89.27 (10.11) | 93.65 (8.58) | 94.62 (12.33) | 0.001 | <0.001 | 88.31 (11.27) | 88.44 (10.9) | 88.76 (10.6) | 92.77 (10.11) | 0.036 | 0.011 | 77.75 (9.8) | 79.06 (9.33) | 79.27 (8.01) | 83.61 (9.5) | 0.003 | 0.001 | 76.66 (7.34) | 79.98 (8.08) | 80.2 (9.1) | 85.31 (10.45) | <0.001 | <0.001 |
| HC, cm, mean (SD) | 99.81 (6.95) | 101.59 (7.79) | 103.76 (8.2) | 104.08 (8.67) | 0.031 | 0.004 | 100.7 (7.92) | 100.79 (8.64) | 100.98 (7.94) | 103.82 (8.3) | 0.074 | 0.023 | 93.16 (6.89) | 93.47 (7.86) | 93.47 (6.67) | 96.46 (6.98) | 0.035 | 0.011 | 93.02 (5.64) | 95.61 (6.65) | 93.58 (7.13) | 97.31 (7.92) | 0.002 | 0.005 |
| WHR, mean (SD) | 0.87 (0.06) | 0.88 (0.06) | 0.9 (0.06) | 0.91 (0.06) | 0.002 | <0.001 | 0.88 (0.07) | 0.88 (0.07) | 0.88 (0.06) | 0.89 (0.07) | 0.355 | 0.112 | 0.83 (0.07) | 0.85 (0.09) | 0.85 (0.06) | 0.87 (0.08) | 0.114 | 0.019 | 0.82 (0.06) | 0.84 (0.05) | 0.86 (0.09) | 0.88 (0.07) | <0.001 | <0.001 |
| SBP, mmHg, mean (SD) | 102.95 (5.5) | 110.92 (3.28) | 116.61 (7.2) | 120.92 (5.37) | <0.001 | <0.001 | 102.15 (7.47) | 111.07 (3.98) | 114.06 (5.51) | 120.77 (5.21) | <0.001 | <0.001 | 100.41 (5.35) | 109.48 (4.58) | 115.51 (6.14) | 121.58 (7.72) | <0.001 | <0.001 | 101 (6.97) | 110.07 (4.96) | 114.42 (5.9) | 120.94 (7.78) | <0.001 | <0.001 |
| DBP, mmHg, mean (SD) | 66.86 (3.97) | 70.61 (1.74) | 76.81 (2.86) | 83.04 (4.74) | <0.001 | <0.001 | 66.35 (4.78) | 71.05 (2.42) | 78.12 (3.04) | 82.96 (4.12) | <0.001 | <0.001 | 66.03 (4.96) | 71.6 (3.05) | 77.19 (2.54) | 82.89 (4.63) | <0.001 | <0.001 | 65.58 (4.5) | 71.97 (3.16) | 77.71 (2.73) | 85.55 (7.27) | <0.001 | <0.001 |
| Hirsutism score, mean (SD) | 2.89 (2.91) | 2.97 (3.03) | 3.11 (2.39) | 3.72 (3.11) | 0.327 | 0.082 | 2.13 (2.47) | 2.45 (2.62) | 3.38 (2.96) | 3.53 (3.01) | 0.072 | 0.002 | 2.41 (2.03) | 2.6 (2.49) | 2.97 (3.2) | 4.06 (2.83) | 0.002 | <0.001 | 2.77 (2.41) | 2.07 (2.08) | 3.25 (3.22) | 3.35 (2.51) | 0.017 | 0.068 |
| Acne score, mean (SD) | 0.57 (0.77) | 0.41 (0.82) | 0.39 (0.69) | 0.46 (0.66) | 0.704 | 0.646 | 0.4 (0.59) | 0.32 (0.66) | 0.32 (0.71) | 0.65 (0.99) | 0.029 | 0.027 | 0.47 (0.88) | 0.5 (0.81) | 0.62 (0.68) | 0.33 (0.69) | 0.319 | 0.38 | 0.57 (0.86) | 0.44 (0.84) | 0.2 (0.52) | 0.31 (0.65) | 0.023 | 0.011 |
| Acanthosis nigricans score, mean (SD) | 1.11 (0.32) | 1.26 (0.48) | 1.25 (0.65) | 1.37 (0.55) | 0.082 | 0.016 | 1.23 (0.53) | 1.27 (0.57) | 1.26 (0.49) | 1.38 (0.67) | 0.427 | 0.136 | 1.02 (0.13) | 1.08 (0.27) | 1.24 (0.5) | 1.14 (0.35) | 0.007 | 0.012 | 1.14 (0.39) | 1.07 (0.25) | 1.15 (0.36) | 1.22 (0.59) | 0.187 | 0.233 |
| Biochemical parameters | | | | | | | | | | | | | | | | | | | | | | | | |
| FBG, mmol/L, mean (SD) | 5.45 (0.59) | 5.30 (0.66) | 5.28 (0.75) | 5.65 (1.21) | 0.075 | 0.078 | 5.21 (0.81) | 5.31 (0.76) | 5.53 (0.93) | 5.58 (0.94) | 0.069 | 0.01 | 4.62 (0.83) | 4.64 (0.73) | 4.59 (0.92) | 4.67 (1.06) | 0.979 | 0.804 | 4.62 (0.67) | 4.73 (0.77) | 4.54 (1.02) | 4.69 (0.9) | 0.587 | 0.999 |
| FINS, pmol/L, mean (SD) | 122.59 (52.88) | 124.79 (69.9) | 127.59 (36.01) | 146.65 (79.5) | 0.132 | 0.029 | 152.32 (159.78) | 145.74 (116.24) | 154.92 (95.4) | 163.23 (120.67) | 0.841 | 0.461 | 47.53 (19.69) | 43 (18.67) | 47.32 (17.99) | 49.91 (21.95) | 0.328 | 0.318 | 43.11 (18.13) | 47.94 (20.47) | 44.81 (16.47) | 50.55 (17.81) | 0.113 | 0.067 |
| HOMA-IR, mean (SD) | 4.32 (2.1) | 4.24 (2.56) | 4.37 (1.64) | 5.41 (3.55) | 0.041 | 0.012 | 5.49 (7.64) | 5.14 (4.95) | 5.6 (3.84) | 6.03 (5.19) | 0.777 | 0.404 | 1.37 (0.54) | 1.29 (0.57) | 1.39 (0.57) | 1.45 (0.59) | 0.515 | 0.334 | 1.27 (0.54) | 1.45 (0.61) | 1.31 (0.55) | 1.53 (0.61) | 0.044 | 0.054 |
| QUICKI, mean (SD) | 0.31 (0.02) | 0.31 (0.02) | 0.31 (0.01) | 0.31 (0.02) | 0.025 | 0.007 | 0.31 (0.02) | 0.31 (0.02) | 0.31 (0.02) | 0.30 (0.02) | 0.074 | 0.01 | 0.37 (0.04) | 0.38 (0.05) | 0.37 (0.03) | 0.37 (0.05) | 0.706 | 0.739 | 0.38 (0.04) | 0.37 (0.03) | 0.38 (0.05) | 0.37 (0.04) | 0.283 | 0.301 |
| HDL, mmol/L, mean (SD) | 1.34 (0.33) | 1.23 (0.37) | 1.29 (0.5) | 1.21 (0.33) | 0.323 | 0.16 | 1.25 (0.33) | 1.2 (0.3) | 1.21 (0.42) | 1.15 (0.33) | 0.509 | 0.189 | 1.43 (0.45) | 1.36 (0.32) | 1.51 (0.45) | 1.28 (0.36) | 0.023 | 0.094 | 1.33 (0.31) | 1.28 (0.37) | 1.29 (0.34) | 1.27 (0.39) | 0.748 | 0.365 |
| LDL, mmol/L, mean (SD) | 3.03 (0.94) | 3.05 (0.97) | 3.53 (1.4) | 3.45 (0.87) | 0.018 | 0.006 | 2.92 (0.76) | 3.13 (0.84) | 2.83 (0.88) | 3.05 (0.82) | 0.212 | 0.946 | 2.83 (0.69) | 2.83 (0.73) | 3.0 (1.04) | 3.08 (0.76) | 0.235 | 0.049 | 2.59 (0.74) | 2.77 (0.82) | 2.69 (0.69) | 2.8 (0.64) | 0.306 | 0.154 |
| TG, mmol/L, mean (SD) | 1.88 (1.04) | 1.75 (0.91) | 2.26 (1.43) | 1.94 (0.8) | 0.106 | 0.34 | 1.98 (0.98) | 1.81 (0.90) | 2.0 (1.11) | 2.03 (1.06) | 0.564 | 0.416 | 1.17 (0.61) | 1.05 (0.42) | 1.1 (0.54) | 1.35 (0.76) | 0.053 | 0.08 | 1.17 (0.65) | 1.17 (0.45) | 1.2 (0.51) | 1.44 (0.82) | 0.072 | 0.032 |
| TC, mmol/L, mean (SD) | 4.89 (1.18) | 4.86 (1.18) | 5.48 (1.74) | 5.29 (1.05) | 0.038 | 0.021 | 4.73 (0.94) | 4.95 (1.06) | 4.58 (1.13) | 4.86 (1.06) | 0.235 | 0.929 | 4.59 (0.89) | 4.54 (0.9) | 4.8 (1.23) | 4.83 (0.94) | 0.31 | 0.097 | 4.27 (0.89) | 4.46 (1.04) | 4.34 (0.87) | 4.55 (0.81) | 0.321 | 0.167 |
| ApoA1, g/L, mean (SD) | 1.57 (0.33) | 1.52 (0.31) | 1.52 (0.33) | 1.53 (0.28) | 0.852 | 0.571 | 1.54 (0.32) | 1.51 (0.32) | 1.46 (0.34) | 1.47 (0.32) | 0.648 | 0.245 | 1.55 (0.35) | 1.51 (0.29) | 1.61 (0.36) | 1.52 (0.34) | 0.524 | 0.866 | 1.47 (0.25) | 1.46 (0.34) | 1.47 (0.3) | 1.49 (0.32) | 0.972 | 0.731 |
| ApoB, g/L, mean (SD) | 0.94 (0.34) | 0.96 (0.32) | 1.12 (0.38) | 1.09 (0.28) | 0.009 | 0.003 | 0.90 (0.26) | 1.00 (0.28) | 0.91 (0.32) | 0.99 (0.26) | 0.116 | 0.328 | 0.79 (0.18) | 0.81 (0.22) | 0.82 (0.27) | 0.90 (0.26) | 0.036 | 0.007 | 0.73 (0.22) | 0.8 (0.22) | 0.78 (0.2) | 0.83 (0.2) | 0.052 | 0.023 |
| ApoB/ApoA1ratio, mean (SD) | 0.61 (0.21) | 0.65 (0.22) | 0.75 (0.24) | 0.73 (0.2) | 0.005 | 0.001 | 0.61 (0.20) | 0.68 (0.21) | 0.63 (0.22) | 0.69 (0.19) | 0.105 | 0.148 | 0.53 (0.16) | 0.56 (0.2) | 0.52 (0.17) | 0.62 (0.19) | 0.029 | 0.017 | 0.51 (0.17) | 0.56 (0.15) | 0.54 (0.15) | 0.58 (0.18) | 0.053 | 0.029 |
| TT, nmol/L, mean (SD) | 2.3 (0.48) | 2.23 (0.55) | 2.15 (0.41) | 2.24 (0.59) | 0.697 | 0.628 | 1.18 (0.32) | 1.19 (0.33) | 1.21 (0.38) | 1.22 (0.29) | 0.921 | 0.493 | 2.16 (0.4) | 2.13 (0.45) | 2.24 (0.47) | 2.23 (0.48) | 0.506 | 0.229 | 1.22 (0.27) | 1.18 (0.32) | 1.19 (0.29) | 1.2 (0.27) | 0.785 | 0.571 |
| FT, pg/ml, mean (SD) | 2.71 (0.72) | 2.79 (0.79) | 2.7 (0.72) | 2.77 (0.73) | 0.925 | 0.874 | 1.93 (0.82) | 2.15 (0.69) | 1.72 (0.69) | 1.99 (0.56) | 0.007 | 0.422 | 2.72 (0.91) | 2.79 (0.74) | 2.56 (0.75) | 2.65 (0.88) | 0.605 | 0.433 | 1.9 (0.67) | 1.85 (0.78) | 1.82 (0.73) | 1.81 (0.62) | 0.882 | 0.187 |
| SHBG, nmol/L, mean (SD) | 37.09 (32.4) | 37.24 (26.68) | 28.81 (21.64) | 31.36 (25.43) | 0.313 | 0.132 | 37.01 (33.36) | 33.37 (19.4) | 42.68 (34.63) | 27.26 (27.91) | 0.023 | 0.129 | 50.89 (32.38) | 47.81 (21.11) | 54.49 (42.87) | 49.19 (30.82) | 0.793 | 0.964 | 53.78 (23.09) | 54.48 (31.87) | 52.75 (31.49) | 49.86 (34.42) | 0.857 | 0.472 |
| FAI, mean (SD) | 9.64 (5.74) | 8.01 (4.33) | 9.19 (3.43) | 10.16 (5.91) | 0.095 | 0.13 | 4.75 (3.28) | 4.71 (2.78) | 4.67 (3.51) | 6.48 (3.61) | 0.002 | 0.001 | 5.99 (3.69) | 5.43 (2.71) | 6.27 (5.34) | 6.42 (4.18) | 0.598 | 0.378 | 2.78 (1.58) | 3.15 (2.85) | 3.25 (2.53) | 3.87 (2.85) | 0.115 | 0.019 |
| LH, mIU/mL, mean (SD) | 12.73 (5.67) | 11.14 (5.47) | 9.84 (4.02) | 10.07 (4.32) | 0.024 | 0.006 | 9.17 (3.88) | 7.59 (4.64) | 8.43 (4.68) | 8.57 (6.55) | 0.416 | 0.899 | 15.16 (7.69) | 12.43 (5.52) | 13.82 (6.32) | 13.44 (5.61) | 0.16 | 0.275 | 10.94 (6.57) | 8.97 (4.71) | 9.81 (5.8) | 9.18 (6.22) | 0.164 | 0.136 |
| FSH, mIU/mL, mean (SD) | 5.93 (1.97) | 5.64 (1.64) | 6.19 (1.65) | 6.03 (1.51) | 0.382 | 0.343 | 6.51 (1.63) | 5.81 (1.89) | 6.16 (1.63) | 5.87 (1.62) | 0.147 | 0.245 | 6.23 (1.48) | 6.32 (1.83) | 6.39 (1.75) | 6.17 (1.31) | 0.905 | 0.826 | 6.09 (1.68) | 6.27 (1.72) | 6.17 (1.83) | 6.25 (1.54) | 0.913 | 0.65 |
| LH/FSH ratio, mean (SD) | 2.61 (2.84) | 2.03 (0.95) | 1.62 (0.66) | 1.72 (0.69) | 0.004 | 0.001 | 1.43 (0.55) | 1.38 (1.06) | 1.36 (0.71) | 1.44 (0.93) | 0.961 | 0.889 | 2.5 (1.35) | 1.99 (0.83) | 2.28 (1.13) | 2.25 (1.01) | 0.128 | 0.426 | 1.86 (1.06) | 1.47 (0.74) | 1.65 (0.93) | 1.51 (1.06) | 0.055 | 0.077 |
| E2, pmol/L, mean (SD) | 294.43 (229.82) | 347.08 (628.02) | 277.93 (200.93) | 277.42 (330.75) | 0.754 | 0.516 | 189.62 (66.31) | 237.96 (302.71) | 221.1 (218.49) | 225.74 (212.8) | 0.761 | 0.678 | 412.21 (539.76) | 301.87 (181.93) | 265.89 (148.32) | 317.27 (324.8) | 0.197 | 0.147 | 262.61 (350.38) | 247.6 (265.84) | 248.18 (167) | 183.98 (70.8) | 0.38 | 0.129 |
| AMH, ng/mL, mean (SD) | 13.15 (5.76) | 13.19 (6.91) | 12.93 (5.26) | 11.64 (6.64) | 0.412 | 0.127 | 11.91 (5.26) | 10.02 (5.36) | 9.59 (4.94) | 8.74 (4.47) | 0.014 | 0.002 | 14.65 (7.06) | 14.58 (6.63) | 14.37 (5.88) | 14.88 (6.39) | 0.985 | 0.868 | 12.98 (6.56) | 10.06 (5.93) | 11.81 (6.24) | 12.36 (7.89) | 0.043 | 0.725 |

BMI, body mass index; WC, waist circumference; HC, hip circumference; WHR, waist-to-hip ratio; SBP, systolic blood pressure; DBP, diastolic blood pressure; MAP, mean arterial pressure; FBG, fasting blood glucose; FINS, fasting insulin; HOMA-IR, homeostatic model assessment-insulin resistance; QUICKI, quantitative insulin sensitivity check index; HDL,high-density lipoprotein; LDL, low-density lipoprotein; TG, triglycerides; TC, total cholesterol; ApoA1, apolipoprotein A1; ApoB, apolipoprotein B; TT, total testosterone; FT, free testosterone; SHBG, sex hormone-binding globulin; FAI, free androgen index; LH, luteinizing hormone; FSH, follicle-stimulating hormone; E2, estradiol; AMH, anti-Müllerian hormon.
